# Machine learning algorithm for early detection of end-stage renal disease

**DOI:** 10.1186/s12882-020-02093-0

**Published:** 2020-11-27

**Authors:** Zvi Segal, Dan Kalifa, Kira Radinsky, Bar Ehrenberg, Guy Elad, Gal Maor, Maor Lewis, Muhammad Tibi, Liat Korn, Gideon Koren

**Affiliations:** 1 Diagnostic Robotics Inc., Ariel, Israel; 2 Ariel University, Ariel, Israel

**Keywords:** End stage renal disease, Machine learning, Prediction model, Algorithm

## Abstract

**Background:**

End stage renal disease (ESRD) describes the most severe stage of chronic kidney disease (CKD), when patients need dialysis or renal transplant. There is often a delay in recognizing, diagnosing, and treating the various etiologies of CKD. The objective of the present study was to employ machine learning algorithms to develop a prediction model for progression to ESRD based on a large-scale multidimensional database.

**Methods:**

This study analyzed 10,000,000 medical insurance claims from 550,000 patient records using a commercial health insurance database. Inclusion criteria were patients over the age of 18 diagnosed with CKD Stages 1–4. We compiled 240 predictor candidates, divided into six feature groups: demographics, chronic conditions, diagnosis and procedure features, medication features, medical costs, and episode counts. We used a feature embedding method based on implementation of the Word2Vec algorithm to further capture temporal information for the three main components of the data: diagnosis, procedures, and medications. For the analysis, we used the gradient boosting tree algorithm (XGBoost implementation).

**Results:**

The C-statistic for the model was 0.93 [(0.916–0.943) 95% confidence interval], with a sensitivity of 0.715 and specificity of 0.958. Positive Predictive Value (PPV) was 0.517, and Negative Predictive Value (NPV) was 0.981. For the top 1 percentile of patients identified by our model, the PPV was 1.0. In addition, for the top 5 percentile of patients identified by our model, the PPV was 0.71.

All the results above were tested on the test data only, and the threshold used to obtain these results was 0.1. Notable features contributing to the model were chronic heart and ischemic heart disease as a comorbidity, patient age, and number of hypertensive crisis events.

**Conclusions:**

When a patient is approaching the threshold of ESRD risk, a warning message can be sent electronically to the physician, who will initiate a referral for a nephrology consultation to ensure an investigation to hasten the establishment of a diagnosis and initiate management and therapy when appropriate.

## Background

End stage renal disease (ESRD) describes the most severe last stage (Stage 5) of chronic kidney disease (CKD), when the kidneys are functioning at 10–15% or less of their normal function [1]. In Stage 1, representing normal renal function, the glomerular filtration rate (GFR) is over 90 ml/kg/min, and the condition is almost always asymptomatic. Stage 2 is defined by GFR between 60 and 89 ml/kg/mi, and although defined by laboratory tests, most individuals are asymptomatic. Stage 3 denotes GFR between 30 and 59 ml/kg/min, and is in most cases associated with fatigue, fluid retention, and changes in urination. Stage 4 is defined by GFR between 15 and 29 ml/kg/min, and is characterized by swelling of the extremities, nausea and vomiting, along with nerve and cognitive malfunction. At Stage 5, the kidneys cannot perform the fluid, electrolyte, and waste exchange needed for homeostasis of the body, and without kidney dialysis or renal transplant, this condition is incompatible with life [2].

Because of the fact that even at Stage 4 persons may be asymptomatic, there is often a delay in recognizing, diagnosing, and treating the various etiologies of CKD. As treatment alternatives exist to slow the progression of renal disease, a precise prediction model is needed for the identification of patients at increased risk for kidney function deterioration [2].

The objective of the present study was to employ machine learning algorithms in an attempt to develop a prediction model for progression to ESRD in patients with CKD, based on a large-scale multidimensional database.

## Methods

### Data set

This study analyzed commercial claims of over 20,000,000 patients from one of the largest United States-based health insurance company from January 1, 2006 to December 31, 2018. The data were selected from medical claims gathered and acquired from their beneficiary’s claims. These data are stored and processed on a regular basis, and it was not gathered specifically for this project. The data were completely de-identified by the insurance company, and all identifying details were removed and were not exposed to the researchers. The medical claims database contains data on medical insurance claims for reimbursement purposes, as well as personal diagnoses according to the International Classification of Diseases, Ninth Revision, Clinical Modification (ICD-9-CM) and International Classification of Diseases, Tenth Revision, Clinical Modification (ICD-10-CM) diagnosis and procedure codes, and details of pharmacy purchases.

### Study population and definitions

This study analyzed 10,000,000 medical insurance claims from 550,000 patient records using a commercial medical claims database. Inclusion criteria were patients over the age of 18 diagnosed with CKD stages 1–4. As the main underlying etiologies for CKD are diabetes and hypertension, patients whose underlying conditions were acute glomerulopathies, congenital abnormalities, or traumatic kidney injury were excluded, as the course of disease in these conditions is different and may interfere with interpretability of the results. The index date for the case group was defined as the date of the first diagnosis of ESRD by a physician (30 ICD-9-CM and ICD-10-CM codes for ESRD, see Additional file 1) or a dialysis procedure. For the control group, the index date was the date of the last available entry in the database, either a diagnosis or a pharmacy purchase. The observation window consisted of all data available 6 months before the index date. Patients who had less than 6 months of claims records prior to index date were excluded.

### Prediction model construction and evaluation

Within the observation window of each patient, we used age, sex, ICD-9-CM and ICD-10-CM diagnostic codes, National Institutes of Health’s RxCUI (RxCUI) medication codes,( [3]- A) and the claims for clinical encounters and costs found in that period for features creation. ICD-9-CM and ICD-10-CM codes were used either directly as diagnostic information in some of the features or by CCS mapping in other features in order to aggregate codes according to medical reasoning. For medication coding, an NDC to RxCUI mapping was done according to NIH conversion tables.

We manually compiled 240 predictor candidates informed by the literature, divided by medical reasoning into six feature groups: demographics, chronic conditions, diagnosis and procedure features, medication features, medical costs, and episode counts.

The index date was calculated individually for each patient as the ESRD diagnosis date for the ESRD positive patients or the date of the last available data for the control. We then left a 6 month prediction window prior to the index date, and generated our features and predictions from only data available prior to the window period.

Chronic condition status was calculated from the claims data using the Center for Medicare and Medicaid Services’ Chronic Condition Data Warehouse (CCW) algorithm standard ( [4]-B). Diagnosis, medications, and procedures features were calculated as count and trend features, and standardized to time of follow-up for the individual patient.

In addition to the manually calculated features, initially inspired by the well-known Word2vec algorithm [5, 6] (a natural language processing method which assigns for each word in a sentence a vector representation), we created an embedding representation (i.e. we converted medical codes into vector representations) for each medical code. The idea was to treat a patient’s set of medical codes as if it were a sentence consisting of words.

**Fig. 1:**
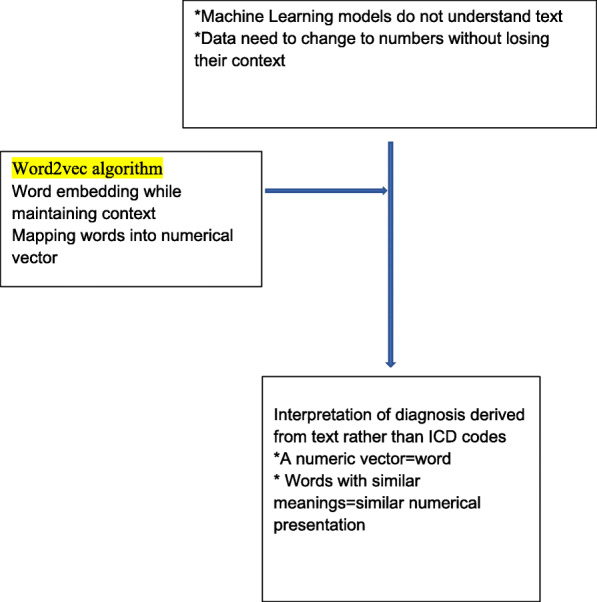
Natural Language Processing with Word2vec algorithm for feature embedding

As claims data do not include direct information on chronic conditions, chronic conditions status was calculated from the claims data. We used the Center for Medicare and Medicaid Services’ Chronic Condition Data Warehouse (CCW) algorithm standard, in which patients are assigned a categorical score of 0 or 1 for each chronic condition according to the prevalence of 1 or more ICD 9 or ICD10 code from a closed medically verified list, within a given timeframe [4, 7]. CCW status was calculated in 3 time frames for each condition - immediate status (3 months before index date), recent (1 year before index date) or ever (any time within the trial window) (Fig. 1).

Next, code embeddings were summed into patient-level vector representations in two different architectures. First, all code embeddings in a patient’s history were summed to form a single patient-level vector. Second, all code embeddings were summed per patient to patient-level vectors During both processes, two types of weights were added per code. The first was Inverse Document Frequency (IDF), which grants higher impact to less frequent codes than frequent ones and, thus, reduces the impact of frequently used administrative codes for example. The second was a temporal weighting function (TWF), which takes into consideration the time interval between the code’s date and the prediction date. In this way, recent codes have more impact than the previous ones. The results of this process were vectors with a length of 100 representing each data component: diagnosis, procedures, and medication.

We treated the prediction of ESRD risk as a binary classification problem. For the analysis, we used the Gradient Boosting trees algorithm (XGBoost implementation) [8].

As the data were imbalanced, the class-weighting version of the XGBoost implementation was used, where the minority represented was over-represented in the algorithm training process in proportion to its’ relative size from the population.

Gradient boosting tree is a machine-learning technique where several decision trees are fit to the data in a stepwise manner where each newly fitted tree is dependent on the previous one, and, thus, an ensemble model is gradually fit so that the prediction loss function is minimized using gradient descent. We randomly divided the cohort into training (development and evaluation of the algorithm’s prediction performance) and testing (evaluating the algorithm’s prediction performance) samples in a ratio of 70:30 (70% training and 30% testing). The model was trained using the training set and the maximum depth of a tree, the minimum child weight, and gamma, as well as the learning rate and the number of trees constructed in the model were tuned by using a 4-fold cross validation procedure. The 4-fold cross validation was implemented on training data only.

In addition, we used a filter method for feature selection. All features with a correlation above 0.9 (correlation coefficient) with another feature (only one from the couple) were excluded. In addition, a deep neural network model (DNN) was examined. This step was implemented on the training data, and then the same selected features were used in the test data. Furthermore, all features that had a normal distribution were normalized using the z-score formula (with the mean and std. of the training set).

Final model results are reported using the testing set by using the best performing model. Optimal model parameters are: max_depth = 6, min_child_weight = 2, n_esitmators = 400, gamma = 0.5, and learning_rate = 0.1.

We compared the XGBoost model with other models, including Logistic Regression with L1 Regularization, Logistic Regression with L2 Regularization, Random Forest and CatBoost.

### Statistical analysis

We compared the patient characteristics by ESRD status and by training and testing samples with unpaired, 2-tailed *t* test, χ^2^ test and analysis of variance, or corresponding nonparametric tests, as appropriate. All analyses were performed using Python, version 3.7 (Python Software Foundation Inc.).

## Results

### Patient characteristics

Beneficiaries in the training (*n* = 19,657) and testing (*n* = 7334) samples had similar characteristics and outcome distributions. The mean [SD] age was 70.72 [± 13.12] years with 50% female patients in the control group, and 70.01 [± 11.95] with 47.1% females in the ESRD patients. The median time to develop ESRD since the starting point of the observation window was 3.35 years.

Clinical factors significantly varied between the case and control groups of patients (Table 1). Positive cases were 1147/19657 (5.8%) of the training population and 438/7334 (6%) in the test population.

**Table 1:** Comparison of calculated features between ESRD positive and ESRD negative patients (performed on all the data)

Feature	Control group ESRD negative Mean (SD) ***n*** = 25,406	Case group ESRD positive Mean (SD) ***n*** = 1585	***p***-value
Acute kidney injury (AKI) episodes (per year)	0.43[±2.01]	0.88[±2.83]	< 0.001
Electrolyte imbalance events (per year)	0.07[±0.41]	0.14[±0.72]	< 0.001
Fluid retention events (per year)	0.01[±0.30]	0.04[±0.56]	< 0.001
Urinalysis exams (per year)	1.39[±1.99]	1.72[±2.50]	< 0.001
Kidney biopsies (per year)	0.001[±0.029]	0.004[±0.050]	< 0.001
Days under ACEi treatment	66.83 ± [107.48]	55.97 ± [93.83]	< 0.001
Hospitalizations	1.10[±2.40]	1.45[±2.83]	< 0.001
Hypertensive crisis episodes (per year)	0.44[±0.99]	1.39[±2.37]	< 0.001
Loop diuretics prescriptions (per year)	0.70[±1.86]	0.97[±1.97]	< 0.001
Lab proteinuria	0.11[±0.51]	0.20[±0.69]	< 0.001
Hyperparathyroidism	0.05[±0.33]	0.13[±0.56]	< 0.001
Phosphorus abnormalities	0.0008[±0.020]	0.0127[±0.215]	< 0.001
Chronic nephritic syndrome	0.001[±0.031]	0.012[±0.19]	< 0.001
Non-nephrogenic complications of diabetes	0.58[±2.16]	0.81[±2.75]	< 0.001
CHF (percent positives)	19.8%	27.3%	< 0.001
Stroke (percent positives)	8.3%	10.5%	0.003
Ischemic heart disease (percent positive)	31.9%	39.3%	< 0.001
Myocardial infarction	36.4%	56.1%	< 0.001
Anemia	35.0%	45.8%	< 0.001
Obesity	18.5%	12.3%	< 0.001
Sex–Female	50%	47.1%	0.08
Sex–Male	50%	52.9%	0.08
Age (years)	70.72 [±13.12]	70.00 [±11.95]	0.83
CKD Stage 1, 6 months before index data	3.67%	0.82%	< 0.001
CKD Stage 2, 6 months before index data	10.00%	3.09%	< 0.001
CKD Stage 3, 6 months before index data	47.04%	35.01%	< 0.001
CKD Stage 4, 6 months before index data	8.14%	52.55%	< 0.001

In the diagnosis and procedures feature group, notable examples of differences between the control and ESRD patients were the count of acute kidney injury (AKI) per year [0.43 vs. 0.88, *p* < 0.001], hypertensive crisis events per year [0.44 vs. 1.39, *p* < 0.001] treatment for electrolyte imbalance per year [0.07 vs. 0.14, *p* value< 0.001], count of fluid retention events per year [0.01 vs. 0.04, *p* < 0.001], number of urinalysis exams per year [1.39 vs. 1.72, *p* < 0.001], and the count of kidney biopsies per year [0.001 vs. 0.004, *p* < 0.001].

In the medication features category, notable examples were annual prescriptions of loop diuretics [0.70 vs. 0.97, *p* < 0.0001]. However, for hypertensive treatment, in a paradoxical fashion ESRD patients spent fewer days under ACEi treatment compared to the control group [66.83 vs. 55.97, *p* < 0.001].

Significant differences were also found in the episode category, where patients had more hospitalizations per year [1.10 vs. 1.45, *p* < 0.001].

**Fig. 2:**
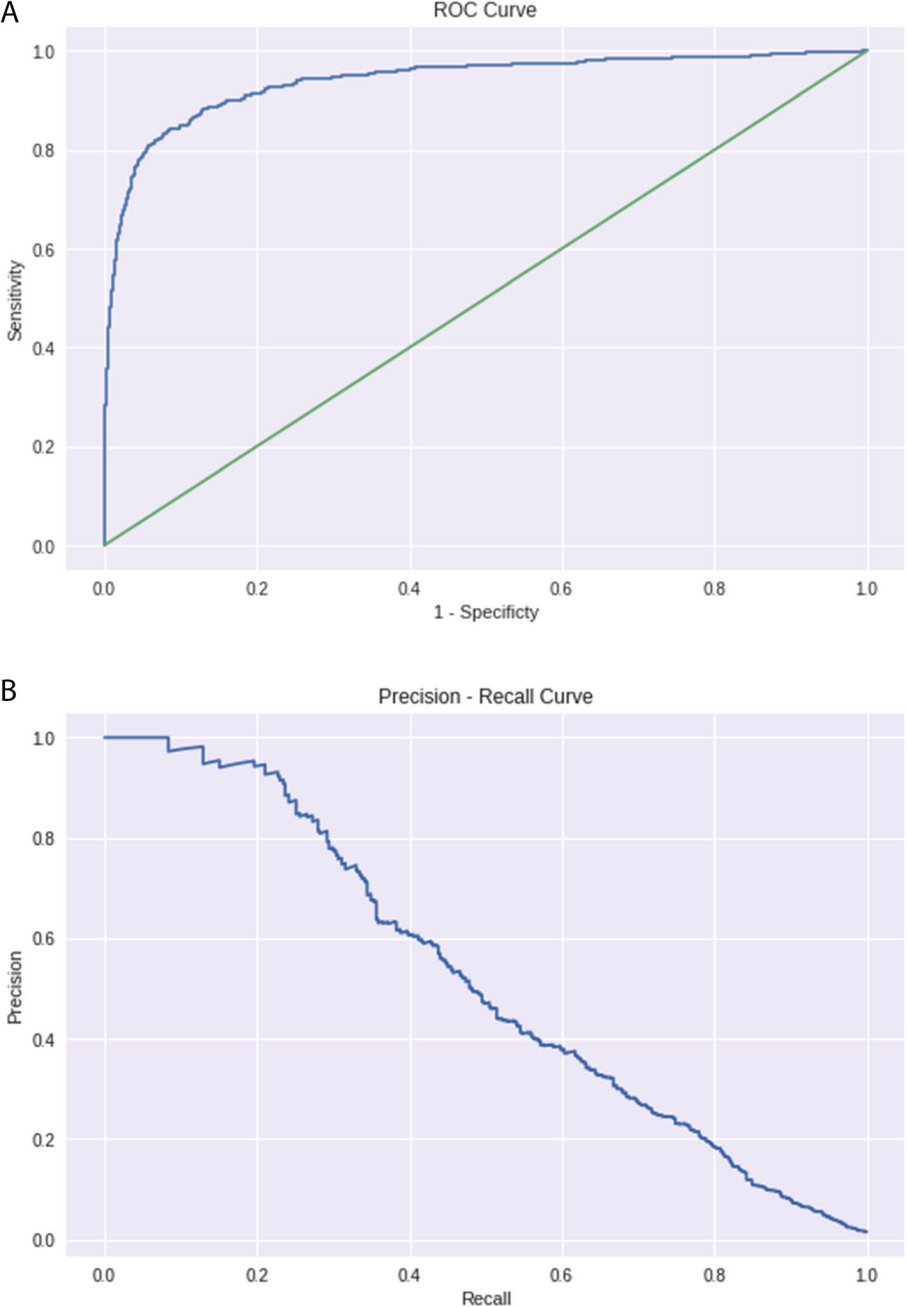
**a**: ROC Curve. Summary of the results of the XGBoost model. The C-statistic for the model was 0.93 (95% confidence intervals for the C-statistic are [0.916–0.943]), with a sensitivity of 0.715 and specificity of 0.958. Positive Predictive Value (PPV) was 0.517 and Negative Predictive Value (NPV) was 0.981. **b**: Precision Recall Curve

Figure 2 summarizes the results of the XGBoost model. The C-statistic for the model was 0.93 (95% confidence intervals for the C-statistic are [0.916–0.943]), with a sensitivity of 0.715 and specificity of 0.958. Positive Predictive Value (PPV) was 0.517 and Negative Predictive Value (NPV) was 0.981. For the top 1 percentile of patients identified by our model, the PPV was 1.0. In addition, for the top 5 percentile of patients identified by our model, the PPV was 0.71. All the results above were tested on the test data only, and the threshold used to obtain these results was 0.1. We estimated the confidence interval by bootstrapping the ROC computations. Notable features contributing to the model were chronic heart failure and ischemic heart disease as a comorbidity, patient age, and number of hypertensive crisis events.

**Fig. 3:**
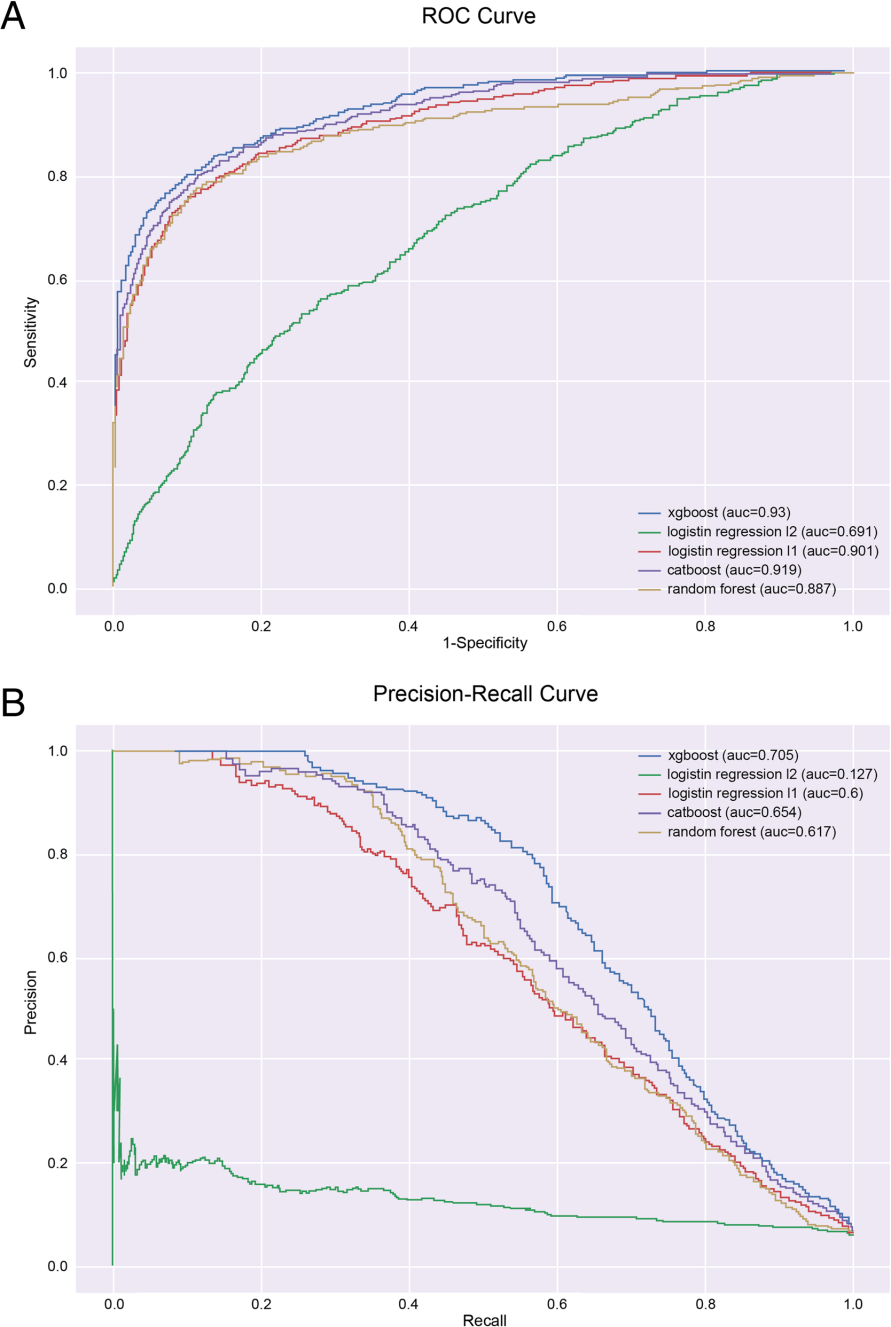
Figure 3 presents different other models that were tested. The c-statistics for the Logistic Regression with L1 Regularization model was 0.901 ([0.884–0.917] 95% confidence interval), with a sensitivity of 0.7 and specificity of 0.928. Besides, the PR-AUC was 0.6 and the F1 score was 0.495. Positive Predictive Value (PPV) was 0.382 and Negative Predictive Value (NPV) was 0.9799. For the top 1 percentile of patients identified by our model, PPV was 0.97. In addition, for the top 5 percentile of patients identified by our model, PPV was 0.62. The threshold used to obtain these results was 0.121. Furthermore, the c-statistics for the CatBoost model was 0.918 ([0.903–0.932] 95% confidence interval), with a sensitivity of 0.7 and specificity of 0.94. Besides, the PR-AUC was 0.653 and the F1 score was 0.53. Positive Predictive Value (PPV) was 0.426 and Negative Predictive Value (NPV) was 0.980. For the top 1 percentile of patients identified by our model, PPV was 0.97. In addition, for the top 5 percentile of patients identified by our model, PPV was 0.66. The threshold used to obtain these results was 0.132. In order to test these models, bounds were chosen according to physicians’ achievement requirements in each model sensitivity (recall) of 0.7–0.8. The figures above show that our model gets better results in other scenarios as well (Fig. 3)

To further investigate the capabilities of our model, a subgroup analysis was carried out (Table 2). Patients were divided into subgroups based on the following criteria: early (Stages 1–2)/ late (Stages 3–4) CKD stage, young (under 60)/older (over 60) years of age, and gender so that each patient was ultimately referenced to one of eight possible different subgroups. The final trained model was implemented on each of the subgroups, as described in Table 2. As shown, optimal results are achieved for young males with early stage disease, and worst results for young males with early stage disease. In general, results are similar for the subgroups, without a significant factor significantly contributing or interfering with model performance.

**Table 2:** Subgroup analysis. Patients were divided into subgroups based on the following criteria: early (Stages 1–2)/ late (Stages 3-4) CKD stage, younger (under 60)/older (over 60) age, and gender so that each patient was ultimately referenced to one of eight possible different subgroups. The final trained model was implemented on each subgroup

	Subgroup size	Positive cases	C- statistics	Sensitivity	Specificity	PPV	NPV
Males ckd S3/S3. Age 60+	1784	164	0.919	0.756	0.931	0.528	0.974
Males,ckd S3/S4, Age 60-	348	44	0.878	0.659	0.908	0.509	0.948
Males ckd S1/S2 Age 60+	1061	16	0.925	0.625	0.983	0.357	0.995
Males ckd S1/S2, Age 60-	559	5	0.968	0.600	0.982	0.231	0.996
Females ckd S3/S4 Age 60+	1862	152	0.918	0.711	0.944	0.529	0.973
Females ckd S3/S4 Age 60-	230	34	0.891	0.765	0.913	0.605	0.957
Females ckd S1/S2 Age 60+	1064	13	0.906	0.765	0.991	0.526	0.997
Females ckd S1/S2 Age 60-	426	10	0.918	0.300	0.993	0.500	0.983

Feature importance analysis (Table 3) performed on the final trained model demonstrated age to be the most important differentiating factor, followed by the highest CKD stage diagnosed during the eligibility period, the annual count of hypertensive crisis diagnosis, and the presence of newly diagnosed (in the past year) hypertension.

**Table 3:** Feature importance analysis

Feature	Feature importance
Age	0.030
CKD stage	0.018
Hypertensive crisis events per year	0.016
Recently diagnosed hypertension	0.013
Total drug prescriptions per year	0.010
Total cost of outpatient specialist visits per year	0.007
Annual medication costs	0.006
Hypertensive nephropathy	0.006
Recently diagnosed hyperlipidemia	0.006
Time gap between last CKD stage diagnosis to most recent	0.004
Number of urinalysis tests per year	0.004
Ever diagnosis of hypertension	0.004
Total cost of ER and inpatient visits per year	0.003
Total annual claims costs	0.003
Acute kidney injury events per year	0.002
Anemia of CKD	0.002
Recently diagnosed diabetes	0.002

This analysis performed on the final trained model demonstrated age to be the most important differentiating factor, followed by the highest CKD stage diagnosed during the eligibility period, the annual count of hypertensive crisis diagnoses, and the presence of newly diagnosed (in the past year) hypertension

We compared the XGBoost model with other models, including Logistic Regression with L1 Regularization, Logistic Regression with L2 Regularization, Random Forest and CatBoost. In addition, a deep neural network model (DNN) was examined. Our model achieved better results in all tested metrics. The following figures display the ROC curve of all models, and the Precision-Recall curve. Figure. 3 in the Additional file 2 shows once more that the XGBoost model achieved the best results in relation to the other models (the blue curve) (Additional file 2).

## Discussion

As ESRD demands kidney dialysis and involves severe comorbidities, accurate prediction of patients who are likely to deteriorate to ESRD at high likelihood of mortality is critical. A variety of methods have been proposed to predict ESRD .

Previous studies have built risk models using logistic or cox regression to predict occurrence of chronic kidney disease (CKD) and its progression in different populations [7]. A number of studies emphasized on building prediction tools for use in patients with CKD, predicting kidney failure (AUC = 0.79 to 0.84), cardiovascular events (AUC = 0.60 to 0.74), and all-cause mortality (AUC = 0.70 to 0.82) [9]. A multinational assessment of risk models for predicting kidney failure in patients with CKD stages 3 to 5 across different geographic regions and patient populations through meta-analysis showed an excellent discrimination across all cohorts with an overall AUC of 0.90 at 2 years and 0.88 at 5 years [10]. Moreover, some existing studies focused on predicting ESRD events in type 2 diabetes patients with AUC ranging from 0.86 to 0.92 for 5-year risk [11–14], while others focused on predicting DKD onset (AUC = 0.68 to 0.72) [15, 16] or major kidney events (e.g., doubling of serum creatinine, renal replacement therapy, or renal death) with AUC of 0.847 [17]. We herein detail some of the methods suggested:

Barret and colleagues set out to determine whether age and comorbidity can be used to predict death within 6 months of the first dialysis in a prospective cohort of 822 patients. No score cutoff was successful in predicting high true-positive and low false-positive rates. Several factors including age, severity of heart failure, arrhythmias, malnutrition, and malignancy were independent prognostic predictors in multivariate models. However, no model was able to accurately predict death within 6 months [18].

Antineutrophil cytoplasmic antibody (ANCA)–associated vasculitides are autoimmune disorders leading to irreversible damage to affected organs. Recently, a new scoring system has been validated as a clinical–pathological method to improve prediction in CKD [19].

Diabetes mellitus is the most common cause of ESRD, leading Wan and colleagues to develop a 5-year ESRD risk prediction model among Chinese patients with type-2 diabetes mellitus in primary care. In a retrospective cohort study, they recruited 149,333 Chinese diabetic adults without ESRD in 2010. Using the cohort over 5 years of follow-up, gender-specific models were derived [13]. The models showed discrimination of 0.866 (males) and 0.862 (females). Age, use of anti-hypertensive drugs, anti drugs, hemoglobin A1c, blood pressure, urine albumin/creatinine ratio (ACR), and estimated glomerular filtration rate (eGFR) were all predictors. Specific predictors for males were smoking and the presence of serious diabetic retinopathy, while important predictors for females included longer duration of diabetes and higher body mass index. Interaction factors included need for insulin and urine ACR in younger males, and eGFR in younger females [13].

The Kidney Failure Risk Equation (KFRE) employs four variables: age, sex, urine albumin-to-creatinine ratio (ACR), and eGFR in individuals with CKD to predict the risk of ESRD and the need for dialysis or a kidney transplant within 2–5 years. In a recent study, Major et al. attempted to validate these predictors [20]. The recalibrated KFRE avidly predicted ESRD risk at 2 and 5 years in primary care. The authors proposed to introduce this model in primary care to reduce unnecessary referrals to secondary care, and earlier referrals for patients who are likely to develop ESRD [20].

Unlike traditional statistics, machine learning tests numerous predictors by combining them in highly interactive computational methods. In the model construction phase, the model generates decision trees aiming to identify success rates of treatment. The model’s success is tested by using 80% of the data for construction and 20% for examination of performance. This process is repeated by dividing the derivation set into new and different learning and testing subsets. The model created by these steps is then applied on previously unused data [21–24].

Our model, based on big data analytics, has shown very high predictive values with c-statistics of 0.93, sensitivity of 0.715, and specificity of 0.958. This model is unique in using insurance claims data. As claims data do not include direct information on chronic conditions, we used the Center for Medicare and Medicaid Services’ Chronic Condition Data Warehouse (CCW) algorithm standard, in which patients are assigned a categorical score for each chronic condition according to the prevalence of ICD 9 or ICD10 code within a given timeframe [4]. CCW status is calculated in 3 time frames for each condition - immediate status (3 months before index date), recent (1 year before index date) or ever (any time within the trial window).

This study has several potential limitations that should be acknowledged. Claims data are restricted to billable elements in the patient’s medical history, often without a clinical context and reasoning. As key information may not be included in claims data, the reliance of our model on the billable ICD9 and ICD10 codes may limit assessment of the correctness of the diagnosis. To overcome the gap that claims data do not include direct information on chronic conditions, we used the Center for Medicare and Medicaid Services’ Chronic Condition Data Warehouse (CCW) algorithm standard, in which patients are assigned a categorical score for each chronic condition according to the prevalence of ICD 9 or ICD10 code within a given timeframe [4]. Future studies should further contrast billing data with other forms of EMR data. Because American EMR data are limited and dispersed among different providers, the much bigger scale of the claims than any other available EMR data, they may increase the overall detection rate of early identification of ESRD. For example, our model could be implemented for stakeholders such as integrated health systems (e.g., health maintenance organizations) where the provider and the payor are inherently linked. In Maccabi Health Services in Israel, an algorithm for early detection of colon cancer based on routine CBC, is linked to patients’ charts and sends the physician a warning to initiate further referrals and diagnostic tests [25]. Similary, it can be used by hospitals and hospital networks linking health care providers with the algorithm based on their claim data.

We used an ensemble tree method (XGBoost) and it can be argued that other methods, such as linear/logistic regression, may be superior. However, when comparing the XGBoost model with other models, including Logistic Regression with L1 Regularization, Logistic Regression with L2 Regularization, Random Forest and CatBoost and deep neural network model (DNN), our model achieved better results in all tested metrics.

## Conclusion

The way this new algorithm may be tested and validated by the stakeholder, for example- health maintenance organization and hospitals, when a patient is approaching the threshold ESRD risk, a warning message can be sent electronically to the physician, to initiate a referral to for a nephrology consultation. An investigation of the specific context of the individual will allow validation, facilitation of a diagnosis and initiation of management when appropriate.

## Supplementary information

**Additional file 1.**

**Additional file 2.**

## Data Availability

Data will become available upon application and approval by the HMO.

## Abbreviations

ESRS: End stage renal disease
CKD: Chronic kidney disease
KFRE: The kidney failure risk equation
ANCA: Antineutrophil cytoplasmic antibody
eGFR: Estimated glomerular filtration rate
