# Supplementary material for: Machine learning algorithm for early detection of end-stage renal disease
Source: BMC Nephrol. 2020 Nov 27;21:518. doi: 10.1186/s12882-020-02093-0 (PMC7693522; doi:10.1186/s12882-020-02093-0)
Supplement: Supplementary file 1 — Additional file 1. [file 12882_2020_2093_MOESM1_ESM.docx]

**Appendix—Machine learning algorithm for early detection of end stage renal disease**

**Table 1: XGBoost model parameters**

Following 4-fold cross-validation hyperparameter tuning, optimal hyperparameters were:

- Max depth=6
- Min child weight=2
- Number of estimators=400
- Gamma=0.5
- Learning rate=0.1
- Positive weight scaling: (length(y_train)-(sum(y_train))/sum(y_train)

The 4-fold cross validation was implemented on the training data only.

Table 2: Codes

| End Stage Renal Disease – Target | | |
| --- | --- | --- |
| ICD-10 Codes | ICD-9 Codes | Description |
| N18.5, N18.6, I12.0, I13.11, I13.2 | 585.5, 585.6, 403.01, 403.11, 403.91, 404.03, 404.13, 404.93, 404.02, 404.12, 404.92 | ESRD |
| Z99.2, Z49, Z49.0, Z49.01, Z49.02, Z49.3, Z49.31, Z49.32 | V45.11, V56.1, V56.2, V56.31, V56.32 | Dependence on / encounter for renal dialysis |
| Current Procedural Terminology (CPT) Codes | | |
| 90935, 90937, 90939, 90940, 90945, 90947, 90989, 90993, 90997, 90999 | | Dialysis |
| 90951-90970 | | End stage renal disease services |

ICD-9 = International Classification of Diseases, Ninth Revision

ICD-10 = International Classification of Diseases, Tenth Revision

| International Classification of Diseases Codes – Inclusion Criteria | | |
| --- | --- | --- |
| Description | ICD-9 Codes | ICD-10 Codes |
| Chronic kidney disease Stage 1–4 | 585.1, 585.2, 585.3, 585.4, 585.9 | N18, N18.1, N18.2, N18.3, N18.4, N18.9 |
| Diabetic nephropathy | 250.40, 250.41, 250.42, 250.43 | E10.21, E10.22, E10.29, E11.2, E11.21, E11.22, E11.29, E13.21, E13.22 |
| Hypertensive nephropathy | 403.00, 403.10, 403.90, 404.00, 404.01, 404.10, 404.11, 404.90, 404.91, 405.01, 405.11, 405.91 | I12, I12.9, I13, I13.0, I13.1, I13.10 I15.0, I15.1 |

| International Classification of Diseases Codes – Exclusion Criteria | | |
| --- | --- | --- |
| ICD-10 Codes | ICD-9 Codes | Description |
| Z94.0, T86.10, T86.11, T86.12, T86.13, T86.19 | V42.0, 996.81 | Kidney transplant |
| N00.0, N00.1, N00.2, N00.3, N00.4, N00.5, N00.6, N00.7, N00.8, N00.9, N01.0, N01.1, N01.2, N01.3, N01.4, N01.5, N01.6, N01.7, N01.8, N01.9, N02.0, N02.1, N02.2, N02.3, N02.4, N02.5, N02.6, N02.7, N02.8, N02.9, N04.0, N04.1, N04.2, N04.3, N04.4, N04.5, N04.6, N04.7, N04.8, N04.9, N05.0, N05.1, N05.2, N05.3, N05.4, N05.5, N05.6, N05.7, N05.8, N05.9, N06.0, N06.1, N06.2, N06.3, N06.4, N06.5, N06.6, N06.7, N06.8, N06.9, N07.0, N07.1, N07.2, N07.3, N07.4, N07.5, N07.6, N07.7, N07.8, N07.9, N08 | 580.0, 580.4, 580.89, 580.9, 581.0, 581.1, 581.2, 581.3, 581.89, 581.9, 583.0, 583.1, 583.2, 583.4, 583.81, 583.89, 583.9 | Acute glomerular nephropathy |
| Current Procedural Terminology (CPT) Codes | | |
| 50327, 50328, 50329, 50340, 50360, 50365 | | Kidney transplant |

| International Classification of Diseases Codes – Symptoms, Diagnosis, and Lab tests | | |
| --- | --- | --- |
| ICD-10 Codes | ICD-9 Codes | Description |
| R630, R634, R636, R64 | 783.0, 783.21, 783.22, 799.4 | Weight loss |
| R110, R1110, R1111, R5382, R8383 | 787.02, 536.2, 787.03, 078.82, 780.71, 780.79 | Nausea and vomiting |
| R569, G4089 | 780.39, 345.80, 345.81 | Convulsions |
| I32, I30.0, I30.8, I30.9 | 420.0, 420.90, 420.91, 420.99, | Pericarditis |
| R37, N521, N52.9, R68.82 | 302.7, 607.84, 799.81 | Sexual dysfunction |
| N91, N92.5, N92.6 | 626 | Irregular menstruation |
| N97.0, N97.8, N79.9 | 628.0, 628.7, 628.8, 628.9 | Female infertility |
| R31 | 599.7, 599.71, 599.72 | Hematuria |
| R34 | 788.5 | Anuria |
| R60.0, R60.9 | 782.3 | Edema |
| E83.3, E83.30, E83.39, E83.5, E83.51, E83.52, E83.59, E87.1, E87.5, E87.7, E87.70, E87.71, E87.79, J90, J91, R18.8 | 275.3, 275.41, 275.42, 275.49, 276.1, 276.61, 276.69, 276.7, 511.9, 789.59 | Fluid and electrolyte abnormalities |
| E21, N25.81, E87.2, E87.8 | 252.0, 252.8, 252.9, 276.2, 588.81 | Disorders of parathyroid gland |
| I15 | 402.01, 402.11, 402.91, 404.00, 404.01, 404.02, 404.10, 404.11, 404.12, 404.90, 404.91, 404.92, 405.01, 405.09, 405.11, 405.19, 405.91, 405.99 | Hypertension |
| I16, I16.0, I16.1, I16.9 |  | Hypertensive crisis |
| D63.1 | 285.21 | Anemia of chronic kidney disease |
| N17 | 583.6, 583.7, 584.5, 584.6, 584.7, 584.8, 584.9 | Acute kidney injury |
| N25.0, N25.8, N25.89 | 588.0, 588.89 | Mineral and bone disorders |
| N03 | 582.0, 582.1, 582.2, 582.4, 582.89, 582.9 | Chronic nephritic syndrome |
| N04 | 581.0, 581.9 | Nephrotic syndrome |
| N10, N11.0, N11.1, N11.8 | 590.00, 590.01, 590.10, 590.11, 593.3,593.4 | Pyelonephritis |
| N11.8, N11.9 | 590.01, 590.8 | Tubulointerstitial nephritis |
| R80.0, R80.1, R80.8, R80.9 | 791.0 | Lab proteinuria |
| R81 | 791.5 | Lab glycosuria |
| R77.0 |  | Lab albumin abnormalities |

| Current Procedural Terminology (CPT) Codes Associated with ESRD | |
| --- | --- |
| CPT Codes | Procedure |
| 93303, 93304 ,93307, 93308, 93312, 93313, 93314, 93315, 93316, 93317, 93318, 93320, 93321, 93325, 93350 ,93501, 93503, 93510, 93511, 93514, 93524, 93526, 93527, 93528, 93529, 93561, 93562 | Cardiac imaging |
| 78700, 78701, 78704, 78707, 78708, 78709, 78710, 78715, 78725 | Renal imaging |
| 81000 ,81001, 81002 ,81003 ,81005, 81007, 81015, 81020, 82040, 82042, 82043, 82044 ,82565, 82570 ,82575, 82668 ,83525, 83527, 84100, 84105 ,80051, 80069 | Urinalysis |
| 50135, 50200, 50205 | Kidney exploration and biopsy |
| 90471, 90472, 90473, 90474 ,90660, 90732, 90740, 90743, 90746, 90747, 90748, 87340 ,87341 ,90371, 80074, 86704, 86705, 86706 | Vaccines |
| 92002, 92004, 92012, 92260 | Eye examination |

| Generic Drug Code Number associated with ESRD | |
| --- | --- |
| RXCUI Codes | RxNorm Class |
| 1808, 62349, 4603, 38413, 2396, 2409, 5487, 5764, 6860, 6916, 167, 3353, 6826, 1001434, 6628, 54365, 644, 214212, 298869, 324042, 258337, 9997, 10763 | Diuretics |
| 1007184, 51428, 1670007, 139825, 274783, 400008, 1605101, 1008501, 86009, 816726, 253182, 1727493, 1858994, 16681, 1368001, 1368384, 1368402, 1373458, 1545149, 2404, 1488564, 1486436, 1727500, 1545653, 1598392, 1664314, 1992672, 1992684, 1992825, 25789, 647235, 4821, 352381, 4815, 285129, 1100699, 1243019, 6809, 607999, 802646, 614348, 1043562, 729717, 30009, 274332, 33738, 73044, 84108, 857974, 593411, 10633, 10635, 1551291, 475968, 1440051, 139953, 1991302 | Treatment for diabetes mellitus |
| 18867, 1998, 3827, 3829, 50166, 29046, 30131, 54552, 35208, 35296, 38454, 1091643, 214354, 83515, 83818, 52175, 321064, 73494, 69749 | Hypertensive control |
| 1895, 1908, 6585, 36118, 11253, 1894, 1313336, 36676 | Electrolytes |
| 105694 | EPO therapy |
