# Supplementary material for: Machine learning algorithm for early detection of end-stage renal disease
Source: BMC Nephrol. 2020 Nov 27;21:518. doi: 10.1186/s12882-020-02093-0 (PMC7693522; doi:10.1186/s12882-020-02093-0)
Supplement: Supplementary file 2 — Additional file 2. [file 12882_2020_2093_MOESM2_ESM.docx]

**APPENDIX:**

Comparison of the XGBoost model with existing models, such as: Logistic Regression with L1 Regularization, Logistic Regression with L2 Regularization, Random Forest and CatBoost. And a deep neural network model (DNN) was problematic in our problem since it tends to overfit.

|  | ROC-AUC | PR-AUC | F1 Score | sensitivity | specificity | Confidence interval for ROC | Precision threshold | PPV | NPV | P@1 | P@5 |
| --- | --- | --- | --- | --- | --- | --- | --- | --- | --- | --- | --- |
| LR L=1 | 0.901 | 0.6 | 0.495 | 0.7 | 0.928 | 0.884 - 0.917 | 0.121 | 0.382 | 0.9799 | 0.97 | 0.62 |
| LR L=2 | 0.691 | 0.127 | 0.16 | 0.7 | 0.562 | 0.666-0.7166 | 0.054 | 0.092 | 0.967 | 0.19 | 0.19 |
| RF | 0.88 | 0.617 | 0.483 | 0.7 | 0.923 | 0.866-0.906 | 0.15 | 0.368 | 0.979 | 0.99 | 0.63 |
| XGBoost | **0.929** | **0.704** | **0.6** | **0.714** | **0.957** | **0.916-0.941** | 0.1 | **0.517** | **0.981** | **1** | **0.71** |
| Catboost | 0.918 | 0.653 | 0.53 | 0.7 | 0.94 | 0.903-0.932 | 0.132 | 0.426 | 0.980 | 0.97 | 0.66 |

Our model achieved better results in all tested metrics.

The following figures display the ROC curve of all models, and the Precision-Recall curve. Figure 3 shows once more that our model (the XGBoost) achieved the best results in relation to the other models (the blue curve).

**Figure 3:**


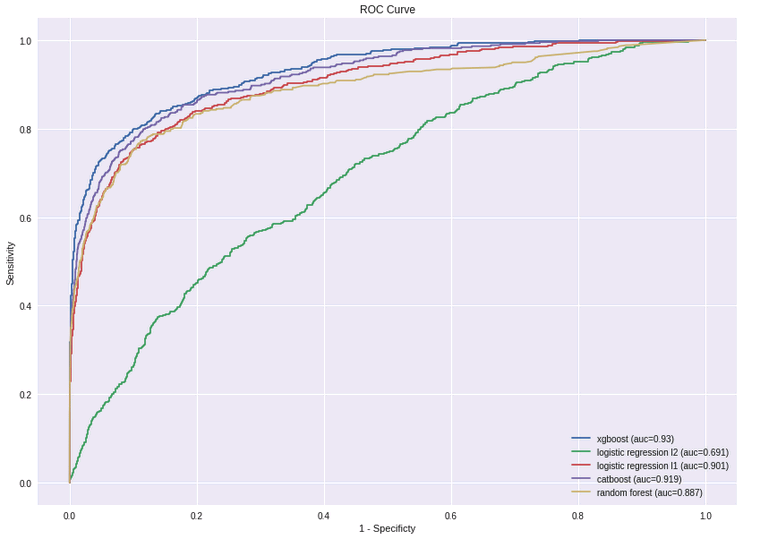


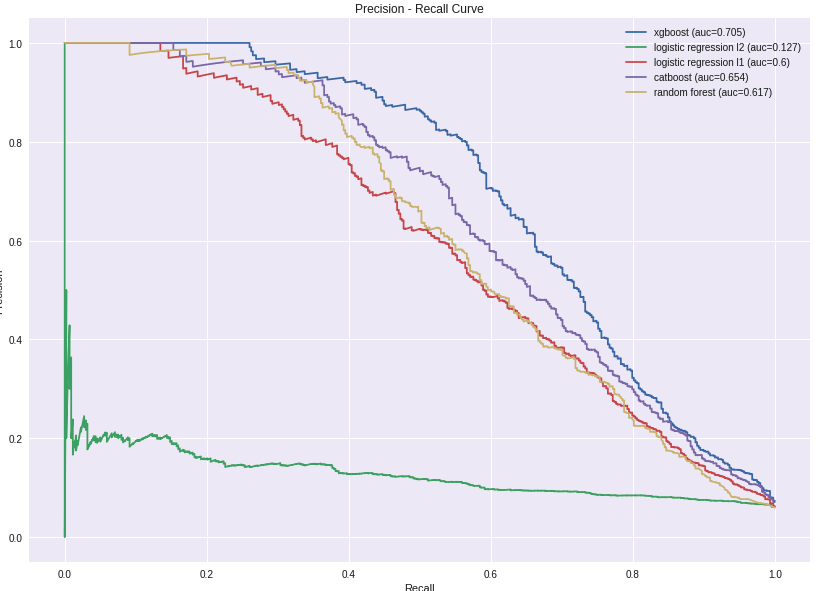


The c-statistics for the Logistic Regression with L1 Regularization model was 0.901 ([0.884 - 0.917] 95% confidence interval), with a sensitivity of 0.7 and specificity of 0.928. Besides, the PR-AUC was 0.6 and the F1 score was 0.495.

Positive Predictive Value (PPV) was 0.382 and Negative Predictive Value (NPV) was 0.9799. For the top 1 percentile of patients identified by our model, PPV was 0.97.

In addition, for the top 5 percentile of patients identified by our model, PPV was 0.62. The threshold used to obtain these results was 0.121.

Furthermore, the c-statistics for the CatBoost model was 0.918 ([0.903-0.932] 95% confidence interval), with a sensitivity of 0.7 and specificity of 0.94.

Besides, the PR-AUC was 0.653 and the F1 score was 0.53.

Positive Predictive Value (PPV) was 0.426 and Negative Predictive Value (NPV) was 0.980. For the top 1 percentile of patients identified by our model, PPV was 0.97.

In addition, for the top 5 percentile of patients identified by our model, PPV was 0.66. The threshold used to obtain these results was 0.132.

In order to test these models, bounds were chosen according to physicians’ achievement requirements in each model sensitivity (recall) of 0.7-0.8. The figures above show that our model gets better results in other scenarios as well.
